# Kidney Tertiary Lymphoid Tissues and Poor Immunosuppressive Response in IgA Nephropathy

**DOI:** 10.1016/j.ekir.2025.11.003

**Published:** 2025-11-17

**Authors:** Hao Zhao, Rui Ma, Zhuoni Zhong, Di Xie, Han Ouyang, Zhanmei Zhou, Cailing Su, Nan Jia, Xin Xu, Fan Fan Hou

**Affiliations:** 1 Division of Nephrology, Nanfang Hospital, Southern Medical University, National Clinical Research Center for Kidney Disease, National Key Laboratory for Prevention and Treatment of Multi-Organ Injury, Guangdong Provincial Institute of Nephrology, Guangdong Provincial Key Laboratory of Renal Failure Research, Guangzhou, China

**Keywords:** IgA nephropathy, immunosuppression, response, tertiary lymphoid tissues

## Abstract

**Introduction:**

Ectopic lymphoid structures termed as tertiary lymphoid tissues (TLTs) are observed in certain kidney diseases, including IgA nephropathy (IgAN); however, clinical relevance of TLTs remains unclear.

**Methods:**

This prospective cohort study included 845 Chinese patients with IgAN with proteinuria ≥ 1 g/d despite 3 months of optimized supportive care with renin-angiotensin system inhibitors. Patients received immunosuppression for a median of 22 months. Kidney interstitial TLTs, defined as lymphocyte aggregates, were counted using immunochemistry and staged by the absence (stage I) or presence of follicular dendritic cells (FDCs) (stage II) or germinal centers (GCs) (stage III). The outcome was response to immunosuppression, defined as no remission with up to 12 months of immunosuppression.

**Results:**

Kidney interstitial TLTs were present in 429 of 845 patients with IgAN (50.8%), of whom 72 of 429 (16.8%) had TLTs at advanced stages (stage II or III). Compared with patients without TLTs, both the presence and stage of TLTs were associated with significantly increased risk of poor response to immunosuppression, with an adjusted odds ratio (95% confidence interval [CI]) of 3.07 (2.16–4.37), 2.97 (2.05–4.28), and 6.25 (3.39–11.53) in those with TLTs at any stage, stage I, and advanced stage, respectively. This grade association remained consistent across all prespecified subgroups. The stage and number of TLTs predicted poor response to immunosuppression with an area under receiver operating characteristic curve (AUC) of 0.81 (95% CI: 0.75–0.87). The AUC increased to 0.90 (95% CI: 0.87–0.94) when we combined TLTs with the clinical and glomerular macrophage data at biopsy.

**Conclusion:**

The presence of interstitial TLTs at diagnosis was associated with poor response to immunosuppression in IgAN.

## Graphical abstract

**Figure:**
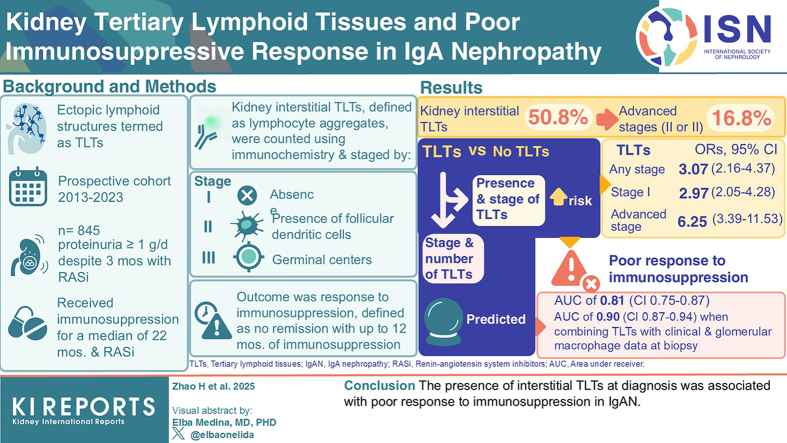

IgAN is the most prevalent primary glomerular disease and progresses to the end-stage kidney disease in about 30% of the patients.^1^ Considering that IgA and complement deposits are always present in kidney, the immune system would be an obvious therapeutic target in those patients. Recently, multiple immunosuppressive strategies, mostly using glucocorticoid and mycophenolate mofetil, have been reported in patients with IgAN who are at high risk of disease progression, with total responders of 52% to 63%.^2^, ^3^, ^4^, ^5^ Therefore, there is a clear need for the early identification of the patients who may not respond to steroid or other immunosuppressive agents, which may help to formulate a more personalized therapy and protect them from the unnecessary exposure to immunologic treatment.

A common feature of IgAN is the infiltration of mononuclear cells in the glomerulus and/or interstitial compartments.^6^^,^^7^ The intensity of cellular inflammation correlates with the severity of kidney histologic lesions, which underlines the rationale for antiinflammatory therapy using corticosteroids or other immunosuppressive agents.^2^, ^3^, ^4^, ^5^^,^^8^ Our recent study found that the intensity of glomerular infiltration with CD206^+^ and CD68^+^ macrophage in the initial diagnostic biopsy specimens was positively associated with the response to immunosuppressive treatment in patients with IgAN, whereas the intensity of tubulointerstitial infiltrates was not.^3^ These findings suggest that intensity and distribution of immune cells in kidney may reflect the local immunologic activity and potentially serves as a marker for predicting response to immunosuppressive therapy.

TLTs are ectopic lymphoid structures consisting mainly of T and B cells and specialized fibroblasts.^9^ TLTs develop within nonlymphoid organs during chronic inflammation conditions such as aging, cancer, autoimmune diseases, and in transplanted organs.^10^, ^11^, ^12^, ^13^, ^14^ The maturation level of TLTs can be classified into 3 stages based on the presence of FDCs and GCs, both represent the cellular components of advanced TLTs.^15^ Though the functional roles of TLTs have not been completely understood, previous studies have reported a strong association between kidney TLTs and maladaptive repair in mice and humans.^16^^,^^17^ Both the presence and the stage of TLTs were reported as an indicator for the severity of kidney injury and a poor prognosis in several kidney diseases, including IgAN.^18^, ^19^, ^20^

To address the question of whether kidney TLTs were associated with poor response to immunosuppressive therapy in patients with IgAN, we analyzed the density and stage of kidney TLTs using immunohistochemical staining in diagnostic biopsy specimens from a prospective cohort of 845 patients with IgAN who were at high risk of disease progression and treated with immunosuppression. We found that the presence of TLTs, at the time of diagnosis, was associated with the poor response to subsequent immunosuppression in patients with IgAN.

## Methods

### Study Design

**Figure 1:**
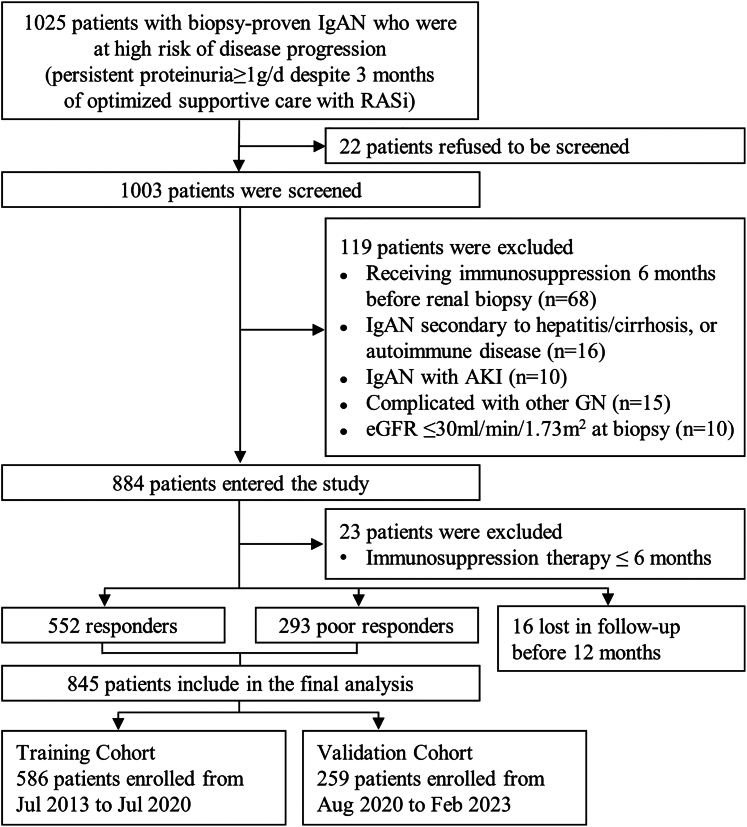
Flowchart of enrollment and exclusion of the study cohorts. AKI, acute kidney injury; eGFR, estimated glomerular filtration rate; GN, glomerulonephritis; IgAN, IgA nephropathy; RASi, renin-angiotensin system inhibitor.

This is a prospective, single-center, cohort study conducted between July 2013 and February 2023 at Nanfang Hospital, a tertiary teaching hospital in Guangzhou, China. A total of 1025 adults with biopsy-proven IgAN who were at high risk of disease progression, that is, those with urinary protein excretion rate ≥ 1.0 g/d despite 3 months of optimized supportive care, were screened for potential participation. The optimized supportive care included maximizing renin-angiotensin system inhibitor,^21^ controlling blood pressure (target systolic blood pressure < 130 mm Hg), and modifying lifestyle.^22^ We excluded the patients with any of the following conditions: (i) IgAN secondary to hepatitis/cirrhosis or autoimmune diseases, (ii) complicated with acute kidney injury or other glomerular diseases, (iii) receiving systemic immunosuppressive agents within 6 months before renal biopsy, and (iv) an estimated glomerular filtration rate (eGFR) < 30 ml/min per 1.73 m^2^ at diagnostic kidney biopsy. The patients were treated with the immunosuppressive agents and renin-angiotensin system inhibitor of choice of the treating nephrologists and followed-up with every 3 months. Among the 884 patients who were enrolled, 845 patients who received ≥ 6 months of immunosuppressive therapy were included in the analysis (Figure 1). The study was approved by the Medical Ethics Committee of Nanfang Hospital. Written informed consent was obtained from all participants of the study.

### Response to the Immunosuppressive Therapy

Complete remission was defined as urinary protein excretion rate decreased to <0.15 g/d with a stable eGFR (< 25% decline from baseline). Partial remission was defined as urinary protein excretion rate decreased by >50% and to a level < 1.0 g/d, and having a stable eGFR. Remission was confirmed by another urine test 30 days later.

Patients with a complete or partial remission within 12 months after the initiation of immunosuppressive therapy were classified as responders, and those failing to achieve above criteria were classified as poor responders.

### Identification and Quantification of Kidney TLTs in IgAN

TLTs in kidney tissue were identified based on the presence of lymphocyte aggregates, as determined by the immunohistochemical staining following the established protocols.^18^^,^^23^ Paraffin-embedded sections of 2-μm–thick were stained using periodic acid-Schiff and scanned with a Digital Pathology Scanner (Leica, Wetzlar, Germany), and the locations of lymphocyte aggregates identified visually.

For each paraffin-embedded kidney tissue, 4 consecutive sections of 4-μm–thick were prepared and stained using immunohistochemistry with the following primary antibodies: mouse antihuman CD3 mAb (M7254; DAKO, Glostrup, Denmark), mouse antihuman CD20 mAb (IR604; DAKO), rabbit antihuman CD21 (ab75985; Abcam, Bristol, UK), and mouse antihuman Ki67 mAb (IR626; DAKO). After incubation with the secondary antibody and visualized using the Envision^+^ System Kit (K5007; DAKO), sections were counterstained with hematoxylin and scanned using a Leica Digital Pathology Slide Scanner (Wetzlar, Germany). Human tonsil tissues were used as the positive control for immunohistochemistry staining, and the sections were incubated with phosphate-buffered saline instead of the primary antibody as the negative control. The TLTs in each section were visually identified as aggregates of ≥60 T cells (CD3^+^) or B cells (CD20^+^) and counted. The density of TLT was calculated as the number of TLTs per 10 mm^2^ cortex.

The TLTs were staged as previously described,^18^ stage I: lacking both FDCs (CD21^+^) and GCs (Ki67^+^); stage II: having FDCs but lacking GCs; and stage III: having both FDCs and GCs. TLTs at stage II or stage III were considered as advanced.

### Measurements of Renal Histologic Lesions and Renal Function

All kidney biopsy specimens were evaluated by 2 independent pathologists who were blinded to the patients' outcomes. The interobserver and intraobserver reproducibility of TLT stage identification was evaluated using intraclass correlation coefficient values. Renal histological lesions were graded according to the Oxford Classification MEST-C score.^24^ The macrophage infiltration in the biopsy specimen was evaluated as previously described.^3^ Urinary protein excretion was quantified in 24-hour urine samples using the biuret method. Serum creatinine levels were measured using the Roche enzymatic method (Hoffmann–La Roche). eGFR was calculated using the Chronic Kidney Disease Epidemiology Collaboration creatinine equation (2009).^25^

### Statistical Analyses

The demographic and clinical characteristics of the patients at biopsy were presented as mean ± SD or median (interquartile range) for continuous variables, and frequency (percentage) for categorical variables. All patients included in the analysis had complete data.

We first used multivariable logistic regression analysis to identify the demographic and the clinical factors at biopsy that were associated with the presence of TLTs. Then, we used logistic regressions to evaluate the association of the stage and the density of TLTs with poor response to immunosuppressive therapy, with adjustment for age, sex, eGFR, 24-hour urine protein (log-transformed), mean arterial pressure, MEST-C score, and the type of immunosuppressive therapy. Both raw and adjusted odds ratios and the corresponding 95% CIs were reported. We used natural cubic splines to estimate the nonlinear relationship between TLT density and the treatment response. We performed subgroup analyses stratified by baseline clinical and histological features, including age (< 35 years, ≥ 35 years), sex, eGFR (< 60, ≥ 60 ml/min per 1.73 m^2^), urinary protein excretion rate (< 1.5, ≥ 1.5 g/d), Oxford T-score (T0, T1, and T2), type of immunosuppressive agents, and use of sodium-glucose cotransporter 2 inhibitors. Further, we performed proportional odds modeling using treatment outcome grades of complete remission, partial remission, and nonresponse to assess potential dose-response relationships. In an additional exploratory analysis, we reclassified the corticosteroid groups into high-dose (> 0.5 mg/kg) and low-dose (≤ 0.5 mg/kg) based on the actual drug doses received and performed a sensitivity analysis using this refined classification.

Furthermore, we divided the study samples into the training set (those enrolled before July 2020) and the validation set (enrolled on or after July 2020), and compared the discrimination performance of different logistic regression models for poor treatment response using AUC as the metrics. The predictive models included univariable models with the presence, the stage, or the density of TLTs. The multivariable model included the following: (i) the clinical variables and the MEST-C scores; (ii) the stages and the density of TLTs; (iii) the stages and the density of TLTs with the clinical variables and the MEST-C scores; (iv) the stages and the density of TLTs with additional incorporation of the infiltration score of glomerular macrophages,^3^ the clinical variables, and the MEST-C scores. Finally, a calibration plot and decision-curve analysis were generated to evaluate the model for the validation cohort.

The results are presented in accordance with the Transparent Reporting of a multivariable Prediction model for Individual Prognosis or Diagnosis guidelines.^26^

Statistical analyses were performed using R version 4.1.3 (R Foundation for Statistical Computing). A 2-sided *P* value < 0.05 was considered statistically significant.

## Results

### Characteristics of the Study Cohort

A total of 845 patients with IgAN who had received ≥ 6 months of immunosuppressive therapy were included in the current study. The characteristics of the study cohort at the time of kidney biopsy and initiation of immunosuppressive therapy, stratified by response to the immunosuppressive therapy, are summarized in Table 1 and Supplementary Table S1, respectively. Among the 845 patients, 166 (19.6%), 238 (28.2%), 408 (48.3%), and 33 (3.9%) received corticosteroids alone, mycophenolate mofetil alone, a combination of corticosteroid and mycophenolate mofetil, and other immunosuppressive agents, respectively. No patient in the study cohort had received rituximab and *Tripterygium wilfordii* Hook. f. Forty-five patients (5.3%) received hydroxychloroquine during follow-up, and 85 patients (10.1%) received sodium-glucose cotransporter 2 inhibitors after their regulatory approval in China in September 2022. The characteristics of the patients at the time of biopsy, stratified by the immunosuppressive agents received, are presented in Supplementary Table S2. In the study cohort, the median (interquartile range) time of immunosuppressive treatment was 22 (15–34) months, and the median (interquartile range) time of follow-up was 27 (22–36) months.

**Table 1:** Characteristic of the patients with IgAN at the time of biopsy

Variables^a^	All participants (*N* = 845)	Responders (*n* = 552)	Poor responders (*n* = 293)
Age, yr	34 ± 10	33 ± 10	36 ± 10
Female, *n* (%)	480 (57)	297 (54)	183 (63)
BMI, kg/m^2^	23 ± 3	23 ± 4	23 ± 4
Smoker, *n* (%)	47 (6)	27 (5)	15 (5)
Hypertension, *n* (%)	145 (17)	90 (16)	52 (18)
History of gross hematuria, *n* (%)	131 (16)	92 (17)	38 (13)
MAP, mm Hg	98 ± 13	97 ± 13	100 ± 14
eGFR, ml/min per 1.73 m^2^	82 ± 29	86 ± 28	75 ± 30
eGFR< 60 ml/min per 1.73 m^2^, *n* (%)	230 (27)	126 (23)	104 (36)
Urinary protein excretion rate, g/d	1.5 (1.1–2.6)	1.5 (1.1–2.5)	1.5 (1.0–2.7)
Serum uric acid, μmol/l	404 ± 113	399 ± 108	413 ± 122
Serum albumin, g/l	36.6 ± 6.3	36.7 ± 6.7	36.4 ± 5.6
Serum triglycerides, mmol/l	1.7 ± 1.2	1.6 ± 1.3	1.8 ± 1.2
Serum cholesterol, mmol/l	5.3 ± 1.8	5.3 ± 1.9	5.2 ± 1.3
Serum LDL-C, mmol/l	3.3 ± 1.3	3.3 ± 1.4	3.2 ± 1.0
Hemoglobin, g/l	128.1 ± 19.6	129.2 ± 19.6	126.1 ± 19.4
Oxford MEST-C, *n* (%)			
M1	777 (92)	504 (91)	273 (93)
E1	157 (19)	101 (18)	56 (19)
S1	767 (91)	501 (91)	266 (91)
T1	298 (35)	187 (34)	111 (38)
T2	146 (17)	70 (13)	76 (26)
C1+2	395 (47)	266 (48)	129 (44)
Presence of TLTs, *n* (%)	429 (51)	223 (40)	206 (70)
Number of TLTs, per 10 mm^2^	0.9 (0–3.9)	0.6 (0–2.3)	3.5 (0–6.2)
Stages of TLTs, *n* (%)			
Stage I	357 (42)	197 (36)	160 (55)
Advanced stage (II+III)	72 (9)	26 (5)	46 (16)
Time from urinary abnormality to renal biopsy, months	23 (16–35)	22 (16–34)	26 (18–39)
Time from biopsy to initiation of immunosuppression, months	3 (2–4)	2 (1–4)	3 (2–4)

BMI, body mass index; eGFR, estimated glomerular filtration rate; IgAN, IgA nephropathy; LDL-C, low-density lipoprotein cholesterol; MAP, mean arterial pressure; MEST-C: (M, mesangial hypercellularity; E, endocapillary hypercellularity; S, segmental glomerulosclerosis; T, interstitial fibrosis/tubular atrophy; C, crescents formation); TLTs, tertiary lymphoid tissues.

a Continuous variables were expressed as mean ± standard deviation or median (25th percentile-75th percentile). Categorical variables were expressed as number (percent).

Within 12 months after initiation of immunosuppressive therapy, 552 patients (65.3%) responded to the treatment, of whom 229 (41.5%) had complete remission. The remission rates, the daily and cumulative dose of the immunosuppressive agents, and the observed adverse events, stratified by the immunosuppressive agents received, are reported in Supplementary Table S3.

### Kidney TLTs and Their Clinical Correlates in the Patients With IgAN

**Figure 2:**
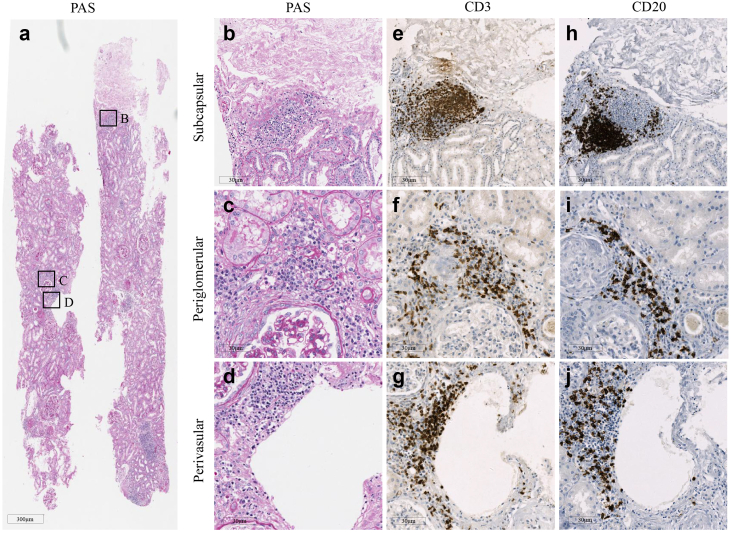
Characteristics of tertiary lymphoid tissues in kidney from patients with IgAN. An overview of PAS-stained kidney tissues from a patient with IgAN revealing multiple lymphocyte infiltrates, as indicated by the (a) boxed areas, which were predominantly located under (b) the kidney capsule, (c) periglomerular region, or (d) surrounding blood vessels, and (e–g) were further confirmed as tertiary lymphoid tissues by CD3 and (h–j) CD20 immunohistochemistry staining. PAS, periodic acid-Schiff.

Multiple mononuclear cell aggregates could be found in either subcapsular, perivascular, or periglomerular areas in periodic acid-Schiff–stained kidney tissues (Figure 2, a–d). These infiltrate structures, confirmed as T and B cells by CD3 and CD20 immunohistochemistry staining, met the definition of TLTs (Figure 2, e–j). Generally, 88.3% of the TLT-like structures identified in periodic acid-Schiff–stained sections were later confirmed as TLTs using immunohistochemistry staining.

**Figure 3:**
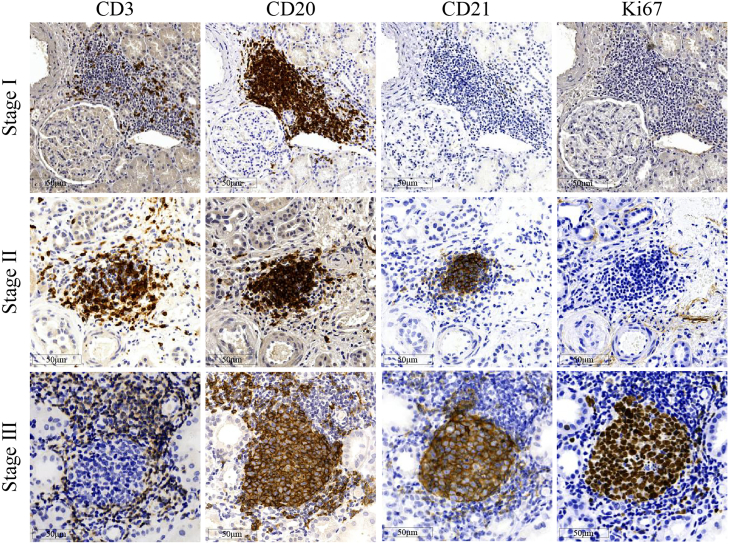
Representative photos of tertiary lymphoid tissues in renal tubulointerstitium in patients with IgA nephropathy. The stages of tertiary lymphoid tissues were determined by the expression patterns of CD3, CD20, CD21, and Ki67 using immunochemistry staining in sequential sections.

An example of the immunohistochemistry staining of the TLTs at different stages is shown in Figure 3. The analysis demonstrated excellent agreement, with an intraclass correlation coefficient of 0.89 (95% CI: 0.84–0.95) for interobserver reproducibility and 0.91 (95% CI: 0.87–0.95) for intraobserver reproducibility. In the study cohort, TLTs were observed in 429 patients (50.8%), of whom 357 (83.2%) were at stage I only and 72 (16.8%) had TLTs at advanced stages (stage II or III).

The characteristics of study cohort at diagnostic biopsy, stratified by TLTs stage, are presented in Supplementary Table S4. In the study cohort, urinary protein excretion rate, tubulointerstitial injury expressed as MEST-C T-score, and time from observed urinary abnormality to kidney biopsy were positively associated with the development of kidney TLTs, whereas eGFR was negatively associated (Table 2).

**Table 2:** Multivariable logistic regression analyses of development of TLTs in the patients with IgAN

Variables	Adjusted OR (95% CI)	*P* value
Age, 10 yrs	1.09 (1.09–1.38)	0.52
Female	1.17 (0.89–1.54)	0.25
BMI, kg/m^2^	1.05 (1.01–1.11)	0.11
MAP, 10 mm Hg	1.02 (0.78–1.03)	0.12
eGFR, 10 ml/min per 1.73 m^2^	0.81 (0.75–0.88)	< 0.001
Urinary protein excretion rate, g/d	1.12 (1.05–1.20)	0.003
Serum uric acid, 10 μmol/l	1.02 (0.99–1.03)	0.16
Serum albumin, 10 g/l	0.86 (0.62–1.12)	0.18
Serum triglycerides, mmol/l	1.04 (0.89–1.18)	0.26
Serum cholesterol, mmol/l	1.08 (0.92–1.23)	0.19
Serum LDL-C, mmol/l	1.12 (0.87–1.43)	0.39
Hemoglobin, 10 g/l	0.99 (0.98–1.01)	0.12
Oxford MEST-C		
M1	1.72 (0.88–3.39)	0.12
E1	1.56 (0.99–2.39)	0.16
S1	2.08 (0.89–3.87)	0.29
T1	3.74 (2.59–5.39)	< 0.001
T2	5.22 (2.98–9.16)	< 0.001
C1+2	1.21 (0.69–1.55)	0.57
Months from urinary abnormality to renal biopsy, mo	1.03 (1.02–1.04)	< 0.001

BMI, body mass index; CI, confidence interval; eGFR, estimated glomerular filtration rate; IgAN, IgA nephropathy; LDL-C, low-density lipoprotein cholesterol; MAP, mean arterial pressure; MEST-C: (M, mesangial hypercellularity; E, endocapillary hypercellularity; S, segmental glomerulosclerosis; T, interstitial fibrosis/tubular atrophy; C, crescents formation); OR, odds ratio; TLTs, tertiary lymphoid tissues.

### The Density and Stage of Kidney TLTs Were Associated With Poor Response to Immunosuppressive Therapy

Presence of kidney TLTs was associated with a 2-fold increase in the adjusted risk of poor response to immunosuppression compared with those without TLTs (Table 3). The relationship between the density of TLTs and poor treatment response appeared to be linear (Supplementary Figure S1). An increase of 1 TLT per 10 mm^2^ section was associated with 41% increase in the risk of poor treatment response. Furthermore, the risk increased with the stage of TLTs, with an adjusted odds ratio (95% CI) of 2.97 (2.05–4.28) for stage I and 6.25 (3.39–11.53) for advanced-stage TLTs.

**Table 3:** The stages and the density of Kidney TLTs correlated with poor response to immunosuppression in the patients with IgAN

	*n*	Proportion responding *n* (%)	Unadjusted OR (95% CI)	*P-*value	Adjusted OR (95% CI)^a^	*P-*value
Presence of TLTs						
No	416	329 (79.1)	Reference	-	Reference	-
Yes	429	223 (52.0)	3.49 (2.58–4.72)	< 0.001	3.07 (2.16–4.37)	< 0.001
Stages of TLTs						
No TLTs	416	329 (79.1)	Reference	-	Reference	-
Stage I	357	197 (55.2)	3.07 (2.24–4.21)	< 0.001	2.97 (2.05–4.28)	< 0.001
Advanced stage (II + III)	72	26 (36.1)	6.69 (3.92–11.43)	< 0.001	6.25 (3.39–11.53)	< 0.001
Density of TLTs, per 10 mm^2^	845	552 (65.3)	1.35 (1.28–1.43)	< 0.001	1.41 (1.31–1.51)	< 0.001

CI, confidence interval; eGFR, estimated glomerular filtration rate; IgAN, IgA nephropathy; OR, odds ratio; TLTs, tertiary lymphoid tissues.

a Adjusted for age, sex, MEST-C score, eGFR, proteinuria, mean arterial pressure and immunosuppressive strategies.

In the subgroup analysis, the association between kidney TLT stage and poor response to immunosuppression was consistent across subgroups stratified by age, sex, clinical variables at the time of kidney biopsy (eGFR and urinary protein excretion rate), Oxford T score, type of immunosuppressive agents, and use of sodium-glucose cotransporter 2 inhibitors (Supplementary Table S5). Higher TLT stage showed a clear monotonic graded association with poorer treatment outcomes (Supplementary Table S6). An additional exploratory analysis, which adjusted for the intensity of the immunosuppressive regimen, further confirmed the association between kidney TLTs and poor response to immunosuppression (Supplementary Table S7).

### Kidney TLTs Improved the Prediction of Poor Response to Immunosuppression in the Patients With IgAN

Among the univariable models, the stages and the density of TLTs predicted poor response to immunosuppression with AUCs of 0.73 (95% CI: 0.68–0.78) and 0.78 (95% CI: 0.73–0.82), respectively. Similar performance was observed in the validation set (Table 4). Both outperformed the multivariable model incorporating the clinical variables and MEST-C scores, which had an AUC of 0.67 (Table 4). The multivariable model with both the density and the stage of TLTs further improved the AUC to 0.81. The full model incorporating the density and the stage of TLTs, the clinical variables, MEST-C scores, and glomerular macrophage infiltration score resulted in a model with the highest AUC at 0.90 (95% CI: 0.87–0.94). The model performance was consistent among the subgroups stratified by the eGFR levels or the type of immunosuppressive agents (Table 5).

**Table 4:** The performance of prediction models for poor response to immunosuppression in the patients with IgAN

Variables	AUC (95% Confidence interval)	
	Training cohort	Validation cohort
TLTs		
Stages of TLTs	0.73 (0.68–0.78)	0.71 (0.65–0.76)
Number of TLTs	0.78 (0.73–0.82)	0.77 (0.71–0.82)
Clinical variables^a^	0.65 (0.61**–**0.69)	0.64 (0.58–0.69)
MEST-C score^b^		
M	0.51 (0.47**–**0.55)	0.51 (0.46**–**0.56)
E	0.50 (0.46**–**0.55)	0.52 (0.47**–**0.57)
S	0.51 (0.45**–**0.56)	0.51 (0.46**–**0.56)
T	0.61 (0.57**–**0.65)	0.61 (0.56**–**0.66)
C	0.52 (0.48**–**0.56)	0.53 (0.48**–**0.57)
Combination		
Clinical variables + MEST-C^c^	0.67 (0.63–0.71)	0.66 (0.60–0.73)
Stages of TLTs + number of TLTs	0.81 (0.75–0.87)	0.79 (0.74–0.85)
Stages of TLTs + number of TLTs + clinical variables + MEST-C^c^	0.83 (0.79–0.86)	0.81 (0.75–0.87)
Stages and number of TLTs + glomerular macrophages^d^ + clinical variables + MEST-C^c^	0.90 (0.87–0.94)	0.89 (0.82–0.96)

AUC, area under the receiver operating characteristic curve; CI, confidence interval; MEST-C: (M, mesangial hypercellularity; E, endocapillary hypercellularity; S, segmental glomerulosclerosis; T, interstitial fibrosis/tubular atrophy; C, crescents formation); TLTs, tertiary lymphoid tissues.

a Clinical variables included age, sex, eGFR, proteinuria, mean arterial pressure at the time of renal biopsy.

b M, mesangial hypercellularity; E, endocapillary hypercellularity; S, segmental glomerulosclerosis; T, interstitial fibrosis/tubular atrophy; C, crescents formation.

c All 5 MEST-C components, each as an independent variable, were included in the model.

d I-score of CD206^+^ and CD68^+^ cells in glomeruli.

**Table 5:** Performance of the predictive model for poor response to immunosuppression in subgroups

Subgroups	Training Cohort		Validation Cohort	
	*n*	AUC (95% CI)	*n*	AUC (95% CI)
Age				
< 35 yrs	338	0.88 (0.84–0.93)	160	0.87 (0.82–0.93)
≥ 35 yrs	248	0.90 (0.85–0.94)	99	0.89 (0.82–0.96)
Sex				
Male	251	0.89 (0.85–0.92)	114	0.88 (0.81–0.95)
Female	335	0.90 (0.86–0.93)	145	0.89 (0.83–0.94)
eGFR				
< 60 ml/min per 1.73 m^2^	175	0.88 (0.83–0.92)	55	0.89 (0.82–0.96)
≥ 60 ml/min per 1.73 m^2^	411	0.91 (0.85–0.96)	204	0.89 (0.84–0.95)
Urinary protein excretion rate				
< 1.5 g/d	329	0.90 (0.84–0.96)	104	0.88 (0.82–0.94)
≥ 1.5 g/d	257	0.89 (0.83–0.94)	155	0.87 (0.82–0.93)
Immunosuppressive agents				
Corticosteroid	124	0.89 (0.83–0.95)	42	0.88 (0.77–0.98)
MMF	180	0.88 (0.84–0.93)	58	0.88 (0.78–0.97)
Corticosteroid and MMF	260	0.90 (0.86–0.94)	148	0.89 (0.82–0.95)
Other immunosuppressive strategies	22	0.87 (0.78–0.96)	11	0.86 (0.72–0.99)

AUC, area under the receiver operating characteristic curve; eGFR, estimated glomerular filtration rate; MMF, mycophenolate mofetil.

The calibration curves demonstrated good agreement between the predicted probabilities and observed outcomes in the validation cohort, as indicated by a Hosmer-Lemeshow test *P*-value of 0.541. Furthermore, decision curve analysis revealed clear clinical utility for the model, showing a superior net benefit compared with the treat-all and treat-none strategies when the threshold probability exceeded approximately 35% (Supplementary Figure S2).

## Discussion

**Figure 4:**
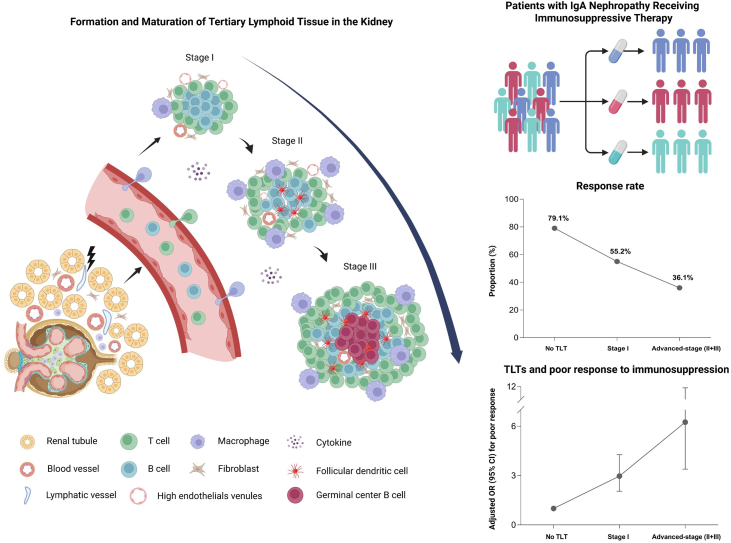
Maturation of kidney tertiary lymphoid tissues (TLT) and association with poor response to immunosuppressive therapy in IgA nephropathy. Schematic representation of kidney TLT maturation and its association with treatment response in patients with IgA nephropathy. The left panel shows morphological features of stage I (immature lymphocyte aggregates), stage II (organized aggregates with follicular dendritic cells), and stage III (advanced structures containing germinal center B cells). The right upper panel illustrates different types of immunosuppressive strategies administered to patients with IgA nephropathy. The right middle panel summarizes the proportion of patients responding to immunosuppressive therapy according to TLT stage (no TLT, stage I, advanced stage II and III). The lower right panel shows the adjusted odds ratio (95% confidence interval) for poor response across TLT stages. (Figure created with biorender.com).

In this large scale, prospective cohort of IgAN, we observed that kidney TLTs were independently associated with the risk of poor response to immunosuppressive therapy in patients with IgAN who were at high risk of disease progression. The predictive model, incorporating kidney TLTs, glomerular macrophage infiltration, and clinical variables, exhibited an excellent performance with an AUC of 0.90. Our data demonstrated the utility of kidney TLTs in identifying the poor responders to immunosuppressive therapy at the time of diagnosis, as schematically depicted in Figure 4.

In our cohort, the kidney interstitial TLTs were observed in 50.8% of the patients with IgAN at the time of diagnosis, which was higher than that reported in a previous study of IgAN (37.5%).^19^ To the best of our knowledge, our study is the first to describe the 3 development stages of TLTs in the kidneys of the patients with IgAN. Kidney TLTs, both the density and their maturity stages, were independently associated with the risk of poor response to immunosuppression. In our study, TLTs significantly outperformed the clinical variables and MEST-C scores in predicting the risk of being poor responders.

If and how TLTs influence the response to immunosuppressive therapy and *vice versa* remain poorly understood. A recent study has demonstrated that TLTs potentially amplify tissue inflammation in aged injured kidneys.^14^^,^^15^ Lymphocytes in TLTs promote proinflammatory phenotypes of the surrounding tubules and fibroblasts via producing proinflammatory cytokines. Furthermore, a reciprocal interaction with interstitial macrophages amplifies this process: macrophage-derived signals promote TLT maturation, whereas advanced TLTs sustain macrophage activation through antigen presentation. These proinflammatory parenchymal cells then interact with immune cells in TLTs by chemokine or cytokine production. Such cell-cell interactions expand TLTs, increase inflammation, exacerbate tissue injury, and promote fibrosis.^14^^,^^15^ The presence of TLTs is associated with a more severe disease course in autoimmune diseases,^27^^,^^28^ and chronic inflammatory diseases, including chronic kidney disease.^19^^,^^29^^,^^30^ These data suggest that kidney TLTs might be a marker reflecting persistency of tissue inflammation and maladaptive fibrotic process, which may be resistant to classical immunosuppression, such as glucocorticoid or mycophenolate mofetil. Potential combined therapy that interferes with the microenvironment alongside targeting immune cells might be rational for treatment of TLT-associated kidney disease such as IgAN.

Kidney immunologic activity is a dynamic process. In the present study, we found that the patients with advance TLTs, as compared with those with stage-I TLTs, had longer duration of disease course and were more prone to disease progression such as kidney dysfunction, severe proteinuria, and tubulointerstitial injury (Supplementary Table S4). These findings are consistent with the previously reported rodent experimental data, in which the proportion of advanced TLTs increased in a time-dependent manner after injury.^15^ Other studies demonstrated that TLTs correlated with the formation of alloantibodies,^31^^,^^32^ and the intensity of antibody-mediated alloimmune responses associated with the maturation status of TLTs.^33^^,^^34^ These results suggest a link between advanced TLTs and kidney damage and provide information for understanding the role of FDCs and GCs; both have been demonstrated to contribute to inflammatory lymphoneogenesis and the diversity of the immune response.^35^^,^^36^

IgAN is characterized by a wide spectrum of histologic lesions, ranging from active and potentially reversible changes that may respond to immunosuppressive agents, to chronic and nonresponsive lesions that are resistant to immunomodulatory therapies.^37^ This variability complicates treatment decisions at the time of diagnosis (kidney biopsy), making it a significant clinical challenge. Moreover, risk factors such as comorbidities, tissue scarring, and hemodynamic overload of the remaining nephrons, associate with disease progression but do not respond to immunomodulatory therapy.^38^ Therefore, biomarkers reflecting kidney immunologic activity are badly needed to identify patients with or without a realistic chance of benefiting from immunomodulation treatment among those with IgAN. Our recent study identified glomerular macrophage infiltration as a good marker for predicting response to immunosuppression in patients with IgAN. The model combining the intensity of glomerular macrophage with clinical and histologic variables showed an excellent predictive performance (AUC: 0.87). Notably, adding the density of TLTs to this model further improved the predictive performance, with AUC increased to 0.90.

A limitation of this study should be mentioned. This is a single-center study conducted and validated in a Chinese cohort. Because the clinical efficacies of immunosuppressive agents in non-Chinese patients have not been demonstrated, validations involving other ethnic populations are warranted. In addition, this study did not include patients receiving novel targeted therapies such as BAFF/APRIL inhibitors or CD38-directed agents, because these drugs have not yet been approved for the treatment of IgAN in China. Future studies that incorporate these therapies in diverse populations are needed.

In conclusion, we found that the density and the stage of kidney TLTs were independently associated with the risk of poor response to immunosuppressive therapy in patients with IgAN who are at high risk of disease progression. The model that combined density of TLTs with glomerular macrophage, MEST-C score and clinical variables had excellent predictive performance, which may help to improve personalized treatment decision in IgAN at the time of kidney biopsy.

## Disclosure

All the authors declared no competing interests.

## Supplementary Material

Supplementary File (PDF) Figure S1. Association between TLT numbers and poor response to immunosuppression in patients with IgAN. Figure S2. Calibration and decision curve analysis of the predictive model in the validation cohort of patients with IgA nephropathy. Table S1. Characteristics of patients with IgAN at initiation of immunosuppressive therapy. Table S2. Characteristics of IgAN patients stratified by immunosuppressive agents at time of renal biopsy. Table S3. The remission rates, daily and cumulative dose and adverse events of immunosuppressive agents in patients with IgAN treated by immunosuppression. Table S4. Characteristics of study cohort according to TLTs stages at diagnostic biopsy. Table S5. Multivariable logistic regression analysis of the association between kidney TLT and poor response to immunosuppression across subgroups. Table S6. Dose-response relationship between kidney TLTs, and poor response to immunosuppression. Table S7. Association between kidney TLT and poor immunosuppressive response in IgAN: sensitivity analysis with dose-stratified regimen categories. STROBE checklist.
