# Supplementary material for: Kidney Tertiary Lymphoid Tissues and Poor Immunosuppressive Response in IgA Nephropathy
Source: Kidney Int Rep. 2025 Nov 17;11(2):103676. doi: 10.1016/j.ekir.2025.11.003 (PMC12769388; doi:10.1016/j.ekir.2025.11.003)
Supplement: Supplementary File (PDF) — Figure S1. Association between TLT numbers and poor response to immunosuppression in patients with IgAN. Figure S2. Calibration and decision curve analysis of the predictive model in the validation cohort of patients with IgA nephropathy. Table S1. Characteristics of patients with IgAN at initiation of immunosuppressive therapy. Table S2. Characteristics of IgAN patients stratified by immunosuppressive agents at time of renal biopsy. Table S3. The remission rates, daily and cumulative dose and adverse events of immunosuppressive agents in patients with IgAN treated by immunosuppression. Table S4. Characteristics of study cohort according to TLTs stages at diagnostic biopsy. Table S5. Multivariable logistic regression analysis of the association between kidney TLT and poor response to immunosuppression across subgroups. Table S6. Dose-response relationship between kidney TLTs, and poor response to immunosuppression. Table S7. Association between kidney TLT and poor immunosuppressive response in IgAN: sensitivity analysis with dose-stratified regimen categories. STROBE checklist. [file mmc1.pdf]

Supplementary Material

Table S1. Characteristic of patients with IgAN at initiation of immunosuppressive therapy

Table S2. Characteristics of IgAN patients stratified by immunosuppressive agents at time of renal biopsy

Table S3. The remission rates, daily and cumulative dose, and adverse events of immunosuppressive agents in patients with IgAN treated by immunosuppression

Table S4. Characteristics of study cohort according to TLTs stages at diagnostic biopsy

Table S5. Multivariable logistic regression analysis of the association between kidney TLT and poor response to immunosuppression across subgroups

Table S6. Dose-response relationship between kidney TLTs, and poor response to immunosuppression

Table S7. Association between kidney TLT and poor immunosuppressive response in IgAN: sensitivity analysis with dose-stratified regimen categories

Figure S1. Association between TLT numbers and poor response to immunosuppression in patients with IgAN

Figure S2. Calibration and decision curve analysis of the predictive model in the validation cohort of patients with IgA nephropathy

STROBE Checklist

Table S1. Characteristic of patients with IgAN at initiation of immunosuppressive therapy

Variables [a]	All participants (n=845)	Responders (n=552)	Poor Responders (n=293)
MAP, mmHg	89 ± 10	88 ± 9	90 ± 10
eGFR, ml/min/1.73 m^2	82 ± 30	86 ± 29	74 ± 31
Urinary protein excretion rate, g/d	1.5 (1.2-2.3)	1.5 (1.2-2.2)	1.5 (1.2-2.4)
Serum albumin, g/L	38.1 ± 5.8	38.2 ± 6.1	37.9 ± 5.1
Serum triglycerides, mmol/L	1.6 ± 0.9	1.6 ± 0.9	1.6 ± 1.0
Serum cholesterol, mmol/L	5.1 ± 1.7	5.2 ± 1.8	5.0 ± 1.3
Serum LDL-C, mmol/L	3.1 ± 1.2	3.1 ± 1.3	3.1 ± 0.9
Hemoglobin, g/L	128.6 ± 19.5	129.4 ± 19.9	127.2 ± 18.6

[a] Continuous variables were expressed as mean ± standard deviation or median (25th percentile-75th percentile). Categorical variables were expressed as number (percent).

Abbreviations: MAP, mean arterial pressure; eGFR, estimated glomerular filtration rate; LDL-C, low-density lipoprotein cholesterol.

Table S2. Characteristics of IgAN patients stratified by immunosuppressive agents at time of kidney biopsy

Variables [a]	Corticosteroids (n=166)	MMF (n=238)	Corticosteroid + MMF (n=408)	Other [b] (n=33)
Age, year	34 ± 10	36 ± 10	33 ± 10	44 ± 12
Female, n (%)	106 (64)	120 (50)	230 (56)	24 (73)
BMI, kg/m^2	23 ± 4	23 ± 4	23 ± 3	25 ± 3
MAP, mmHg	97 ± 14	100 ± 13	98 ±12	101 ± 17
eGFR, ml/min/1.73 m^2	91 ± 29	75 ± 29	83 ± 28	83 ± 28
Urinary protein excretion rate, g/d	1.5 (1.0-2.8)	1.5 (1.1-2.0)	1.6 (1.1-2.7)	1.6 (1.2-2.5)
Serum uric acid, µmol/L	408 ± 109	415 ± 114	403 ± 113	404 ± 107
Serum albumin, g/L	35.9 ± 8.4	36.2 ± 4.8	36.3 ± 5.8	35.7 ± 6.4
Serum triglycerides, mmol/L	1.6 ± 1.0	1.6 ± 1.1	1.7 ± 1.1	1.8 ± 0.8
Serum cholesterol, mmol/L	5.8 ± 2.5	4.9 ± 1.1	5.3 ± 1.6	5.7 ± 2.0
Serum LDL-C, mmol/L	3.6 ± 1.8	3.4 ± 0.8	3.3 ± 1.2	3.5 ± 1.3
Hemoglobin, g/L	128.8 ± 19.5	128.5 ± 19.6	127.4 ± 19.7	129.7 ± 18.8
Oxford MEST-C, n (%)				
M1	147 (89)	218 (92)	381 (93)	31 (94)
E1	25 (15)	38 (16)	88 (22)	6 (18)
S1	135 (81)	223 (94)	384 (94)	25 (76)
T1	49 (30)	78 (33)	159 (39)	12 (36)
T2	18 (11)	59 (25)	65 (16)	4 (12)
C1+2	51 (31)	106 (45)	225 (55)	13 (39)

[a] Continuous variables were expressed as mean ± standard deviation or median (25th percentile-75th percentile). Categorical variables were expressed as number (percent).

[b] Other immunosuppressive treatment included tacrolimus and cyclosporine.

Abbreviations: BMI, body mass index; MAP, mean arterial pressure; eGFR, estimated glomerular filtration rate; LDL-C, low-density lipoprotein cholesterol; MEST-C: M, mesangial hypercellularity; E, endocapillary hypercellularity; S, segmental glomerulosclerosis; T, interstitial fibrosis/tubular atrophy; C, crescents formation; MMF: mycophenolate mofetil.

Table S3. The remission rates, daily and cumulative dose, and adverse events of immunosuppressive agents in patients with IgAN treated by immunosuppression

Immunosuppression	n	Daily dose	Cumulative dose	Total remission No. (%)	Complete remission No. (%)	Adverse Events [b] No. (%)
Corticosteroids	166	35 ± 12.4 mg	9228 ± 3856 mg	112 (67.5)	46 (27.7)	7 (4.2)
MMF	238	1.3 ± 0.2 g	867 ± 203 g	154 (64.7)	63 (26.5)	10 (4.2)
Corticosteroids + MMF	408	Steroid 26 ± 9.9 mg, MMF 1.3 ± 0.3 g	Steroid 8370 ± 2635 mg, MMF 895 ± 226 g	265 (65.0)	111 (27.2)	21 (5.1)
Other [a]	33	-	-	21 (63.6)	9 (27.3)	2 (6.1)

[a] Other immunosuppressive treatment included tacrolimus and cyclosporine.

[b] Adverse events included infections (n=19, including upper respiratory tract infection [n=12], skin infection [n=5], and herpes zoster [n=2]), gastrointestinal syndromes such as abdominal distension, increased stool frequency (n=4), newly diagnosed diabetes (n=5) and elevated transaminase (n=12).

Abbreviations: MMF, mycophenolate mofetil.

Table S4. Characteristics of study cohort according to TLTs stages at diagnostic biopsy

**Variables** [a]	**No TLTs (n=416)**	**Stage I TLTs (n=357)**	**Advanced-stage TLTs (n=72)**
Age, year	33 ± 10	35 ± 10	37 ± 10
Female, n (%)	227 (55)	211 (59)	42 (58)
BMI, kg/m^2	23 ± 4	23 ± 4	23 ± 3
MAP, mmHg	98 ± 13	99 ± 12	101 ± 14
eGFR, ml/min/1.73 m^2	94 ± 25	74 ± 28	59 ± 27
Urinary protein excretion rate, g/d	1.3 (1.0-2.3)	1.6 (1.1-2.6)	2.1 (1.3-3.1)
Serum uric acid, µmol/L	381 ± 104	423 ± 119	443 ± 108
Serum albumin, g/L	37.3 ± 7.0	36.2 ± 5.3	34.4 ± 5.9
Serum triglycerides, mmol/L	1.6 ± 1.3	1.7 ± 1.1	2.0 ± 1.4
Serum cholesterol, mmol/L	5.4 ± 2.1	5.1 ± 1.2	5.4 ± 1.4
Serum LDL-C, mmol/L	3.4 ± 1.6	3.2 ± 0.9	3.4 ± 1.1
Hemoglobin, g/L	131.9 ± 19.5	125.0 ± 18.6	121.9 ± 20.9
Oxford MEST-C, n (%)			
M1	363 (87)	343 (96)	71 (99)
E1	64 (15)	75 (21)	18 (25)
S1	362 (87)	337 (94)	68 (94)
T1	101 (24)	168 (47)	29 (40)
T2	28 (7)	84 (24)	34 (47)
C1+2	181 (44)	177 (50)	37 (51)
Time from urinary abnormality to renal biopsy, month	18 (11-26)	24 (18-31)	29 (24-35)

[a] Continuous variables were expressed as mean ± standard deviation or median (25th percentile-75th percentile). Categorical variables were expressed as number (percent).

Abbreviations: BMI, body mass index; MAP, mean arterial pressure; eGFR, estimated glomerular filtration rate; LDL-C, low-density lipoprotein cholesterol; MEST-C: M, mesangial hypercellularity; E, endocapillary hypercellularity; S, segmental glomerulosclerosis; T, interstitial fibrosis/tubular atrophy; C, crescents formation; TLTs, tertiary lymphoid tissues.

Table S5. Multivariable logistic regression analysis of the association between kidney TLT and poor response to immunosuppression across subgroups

Subgroups	Proportion responding n (%)	Stages of TLTs			Density of TLTs, per 10mm^2 (OR, 95%CI)	P for interaction [b]	P for interaction [c]	P for interaction [d]
		No TLTs	Stage I (OR, 95%CI)	Advanced-stage (II+III) (OR, 95%CI)				
Age						0.501	0.338	0.275
<35	342/498 (68.67)	Reference	2.29 (1.35-4.14)	5.52 (2.01-9.05)	1.38 (1.27-1.51)			
≥35	210/347 (60.52)	Reference	2.93 (1.32-5.17)	7.72 (3.35-11.39)	1.35 (1.22-1.50)			
Sex						0.637	0.522	0.585
Male	255/365 (69.86)	Reference	2.23 (1.28-3.89)	4.37 (1.40-8.13)	1.36 (1.23-1.51)			
Female	297/480 (61.88)	Reference	3.11 (2.02-5.10)	8.21 (3.47-12.01)	1.37 (1.26-1.50)			
eGFR						0.516	0.491	0.563
<60 ml/min/1.73m^2	128/233 (54.94)	Reference	2.26 (1.38-4.32)	7.87 (2.98-10.91)	1.37 (1.24-1.52)			
≥60 ml/min/1.73m^2	424/612 (69.28)	Reference	3.21 (2.15-4.79)	5.63 (2.11-8.77)	1.39 (1.27-1.52)			
Urinary protein excretion rate						0.479	0.538	0.273
<1.5g/d	284/433 (65.59)	Reference	3.68 (2.24-6.05)	5.34 (2.06-13.89)	1.42 (1.28-1.58)			
≥1.5 g/d	268/412 (65.05)	Reference	2.19 (1.28-3.73)	5.31 (2.40-11.75)	1.36 (1.24-1.48)			
Oxford T-score						0.473	0.335	0.943
T0	295/401 (73.57)	Reference	2.46 (1.49-4.06)	3.67 (1.39-7.14)	1.32 (1.17-1.50)			
T1	187/298 (62.75)	Reference	2.59 (1.47-4.56)	5.66 (2.31-13.85)	1.37 (1.24-1.51)			
T2	70/146 (47.95)	Reference	3.48 (1.18-9.27)	8.84 (3.19-18.06)	1.37 (1.20-1.55)			
Immunosuppressive agents [a]						0.341	0.313	0.267
Corticosteroid	112/166 (67.47)	Reference	3.73 (1.57-8.83)	6.76 (1.57-21.02)	1.49 (1.24-1.78)			
MMF	154/238 (64.71)	Reference	3.61 (1.33-7.15)	7.44 (3.35-13.18)	1.42 (1.24-1.62)			
Corticosteroid and MMF	265/408 (64.95)	Reference	3.38 (1.98-5.78)	5.57 (2.41-10.87)	1.36 (1.24-1.49)			
SGLT2 inhibitors						0.832	0.856	0.778
No	490/760 (64.47)	Reference	2.88 (2.02-4.12)	6.14 (3.32-11.35)	1.38 (1.29-1.48)			
Yes	62/85 (72.94)	Reference	2.63 (0.83-8.27)	3.94 (0.66-23.68)	1.31 (1.11-1.54)			

[a] Patients receiving other immunosuppressive agents were not analyzed, owing to the limited number of such patients.
[b] Between subgroups and TLT stage I
[c] Between subgroups and advanced-stage (II+III)
[d] Between subgroups and TLT density
Abbreviations: TLTs, Tertiary lymphoid tissues; OR, Odds ratio; CI, Confidence interval.

Table S6. Dose-response relationship between kidney TLTs and poor response to immunosuppression [a]

Stages of TLTs	Proportion responding			Unadjusted OR (95%CI)	*P* value	Adjusted OR (95%CI)	*P* value
	CR	PR	NR				
No TLTs	185 (44.5)	144 (34.6)	87 (20.9)	Reference	-	Reference	-
Stage I	80 (22.4)	117 (32.8)	160 (44.8)	2.94 (2.24-3.85)	<0.001	2.56 (1.89-3.46)	<0.001
Advanced-stage (II+III)	6 (8.3)	20 (27.8)	46 (63.9)	6.81 (4.11-11.33)	<0.001	5.90 (3.39-10.25)	<0.001

[a] Treatment response in this analysis refers to graded outcome categories of complete remission, partial remission, and poor response.

Abbreviations: TLTs, Tertiary lymphoid tissues; OR, Odds ratio; CI, Confidence interval; CR, complete remission; PR, partial remission.

Table S7. Association between kidney TLT and poor immunosuppressive response in IgAN: sensitivity analysis with dose-stratified regimen categories

	n	Proportion responding n (%)	Unadjusted OR (95% CI)	*P* value	Adjusted OR (95% CI) [a]	*P* value
**Presence of TLTs**						
No	416	329 (79.1)	Reference	-	Reference	-
Yes	429	223 (52.0)	3.49 (2.58-4.72)	<0.001	3.05 (2.14-4.36)	<0.001
**Stages of TLTs**						
No TLTs	416	329 (79.1)	Reference	-	Reference	-
Stage I	357	197 (55.2)	3.07 (2.24-4.21)	<0.001	2.96 (2.03-4.21)	<0.001
Advanced-stage (II+III)	72	26 (36.1)	6.69 (3.92-11.43)	<0.001	6.23 (3.36-11.50)	<0.001
**Density of TLTs, per 10mm^2**	845	552 (65.3)	1.35 (1.28-1.43)	<0.001	1.40 (1.30-1.51)	<0.001

[a] Adjusted for age, sex, MEST-C score, eGFR, proteinuria, mean arterial pressure, type and intensity of immunosuppressive strategies.

Abbreviations: TLTs, Tertiary lymphoid tissues; OR, Odds ratio; CI, Confidence interval.

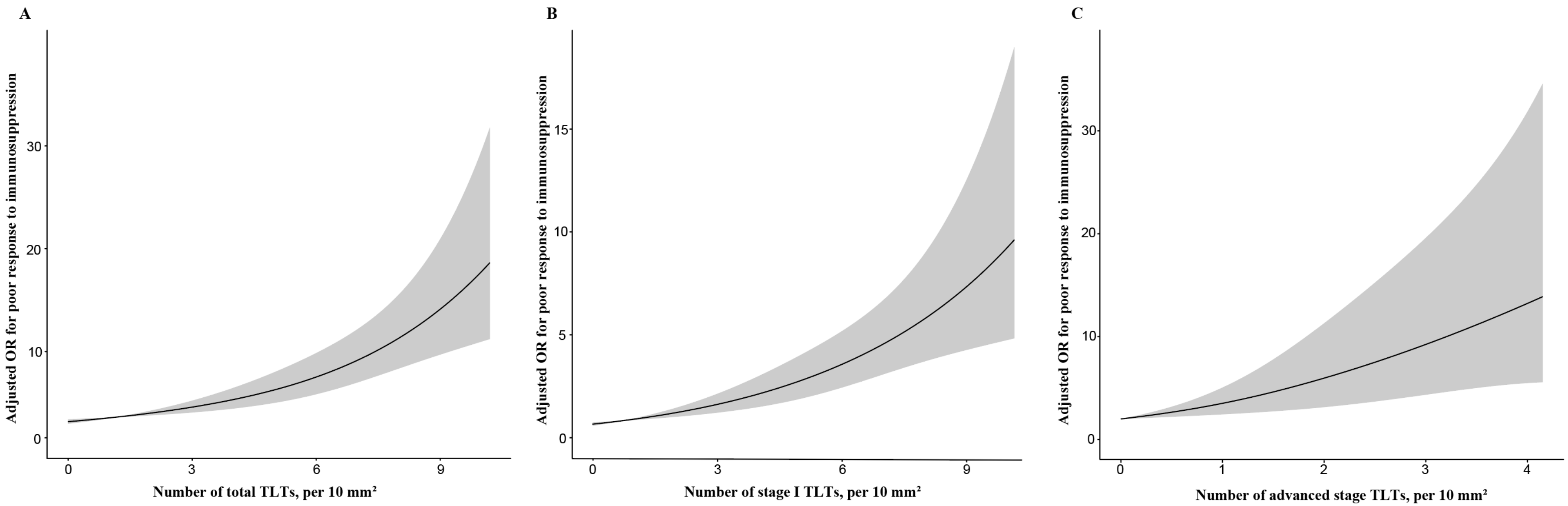

Figure S1. Association between TLT numbers and poor response to immunosuppression in patients with IgAN. Restricted cubic spline analysis of the relationship between numbers of total (A), stage I (B), and advanced-stage (C) TLTs and poor response to immunosuppression in patients with IgAN. Odds ratios (ORs) were adjusted for age, sex, MEST-C score, eGFR, proteinuria, mean arterial pressure, and immunosuppressive strategies.

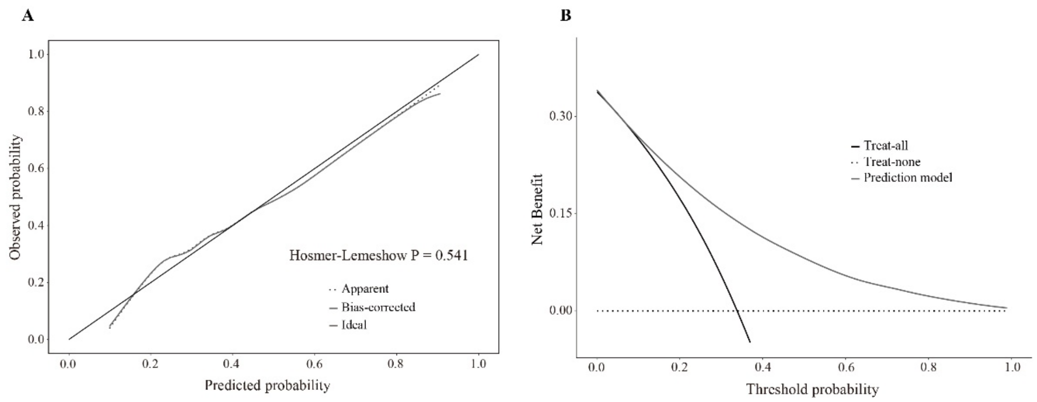

Figure S2. Calibration and decision curve analysis of the predictive model in the validation cohort of patients with IgA nephropathy.

(A) Calibration curve. (B) Decision curve analysis. The model includes the density and stage of TLTs, clinical variables, MEST-C scores, and the glomerular macrophage infiltration score.

STROBE Statement—Checklist of items that should be included in reports of ***cohort studies***

	Item No	Recommendation	Page No
**Title and abstract**	1	(*a*) Indicate the study's design with a commonly used term in the title or the abstract<br>(*b*) Provide in the abstract an informative and balanced summary of what was done and what was found	1-3
**Introduction**			
Background/rationale	2	Explain the scientific background and rationale for the investigation being reported	4-5
Objectives	3	State specific objectives, including any prespecified hypotheses	5
**Methods**			
Study design	4	Present key elements of study design early in the paper	6
Setting	5	Describe the setting, locations, and relevant dates, including periods of recruitment, exposure, follow-up, and data collection	6
Participants	6	(*a*) Give the eligibility criteria, and the sources and methods of selection of participants. Describe methods of follow-up<br>(*b*) For matched studies, give matching criteria and number of exposed and unexposed	6
Variables	7	Clearly define all outcomes, exposures, predictors, potential confounders, and effect modifiers. Give diagnostic criteria, if applicable	7-9
Data sources/ measurement	8*	For each variable of interest, give sources of data and details of methods of assessment (measurement). Describe comparability of assessment methods if there is more than one group	7-9
Bias	9	Describe any efforts to address potential sources of bias	9-11
Study size	10	Explain how the study size was arrived at	6
Quantitative variables	11	Explain how quantitative variables were handled in the analyses. If applicable, describe which groupings were chosen and why	9-11
Statistical methods	12	(*a*) Describe all statistical methods, including those used to control for confounding<br>(*b*) Describe any methods used to examine subgroups and interactions<br>(*c*) Explain how missing data were addressed<br>(*d*) If applicable, explain how loss to follow-up was addressed<br>(*e*) Describe any sensitivity analyses	9-11
**Results**			
Participants	13*	(a) Report numbers of individuals at each stage of study—eg numbers potentially eligible, examined for eligibility, confirmed eligible, included in the study, completing follow-up, and analysed<br>(b) Give reasons for non-participation at each stage<br>(c) Consider use of a flow diagram	11-12
Descriptive data	14*	(a) Give characteristics of study participants (eg demographic, clinical, social) and information on exposures and potential confounders<br>(b) Indicate number of participants with missing data for each variable of interest<br>(c) Summarise follow-up time (eg, average and total amount)	11-12
Outcome data	15*	Report numbers of outcome events or summary measures over time	12

Main results	16	(*a*) Give unadjusted estimates and, if applicable, confounder-adjusted estimates and their precision (eg, 95% confidence interval). Make clear which confounders were adjusted for and why they were included<br>(*b*) Report category boundaries when continuous variables were categorized<br>(*c*) If relevant, consider translating estimates of relative risk into absolute risk for a meaningful time period	14-15
Other analyses	17	Report other analyses done—eg analyses of subgroups and interactions, and sensitivity analyses	14
**Discussion**			
Key results	18	Summarise key results with reference to study objectives	16
Limitations	19	Discuss limitations of the study, taking into account sources of potential bias or imprecision. Discuss both direction and magnitude of any potential bias	19
Interpretation	20	Give a cautious overall interpretation of results considering objectives, limitations, multiplicity of analyses, results from similar studies, and other relevant evidence	16-18
Generalisability	21	Discuss the generalisability (external validity) of the study results	19
**Other information**			
Funding	22	Give the source of funding and the role of the funders for the present study and, if applicable, for the original study on which the present article is based	20

*Give information separately for exposed and unexposed groups.

Note: An Explanation and Elaboration article discusses each checklist item and gives methodological background and published examples of transparent reporting. The STROBE checklist is best used in conjunction with this article (freely available on the Web sites of PLoS Medicine at http://www.plosmedicine.org/, Annals of Internal Medicine at http://www.annals.org/, and Epidemiology at http://www.epidem.com/). Information on the STROBE Initiative is available at http://www.strobe-statement.org.
